# LMP7 as a Target for Coronavirus Therapy: Inhibition by Ixazomib and Interaction with SARS-CoV-2 Proteins Nsp13 and Nsp16

**DOI:** 10.3390/pathogens14090871

**Published:** 2025-09-02

**Authors:** Yi Ru, Yue Ma-Lauer, Chengyu Xiang, Pengyuan Li, Brigitte von Brunn, Anja Richter, Christian Drosten, Andreas Pichlmair, Susanne Pfefferle, Markus Klein, Robert D. Damoiseaux, Ulrich A. K. Betz, Albrecht von Brunn

**Affiliations:** 1 Max-von-Pettenkofer Institute, Virology Department, Ludwig-Maximilians-University of Munich, 80336 Munich, Germany; ru@mvp.lmu.de (Y.R.); ma_lauer@mvp.lmu.de (Y.M.-L.); xiang@mvp.lmu.de (C.X.); leepy1022@outlook.com (P.L.); vonbrunn_b@mvp.lmu.de (B.v.B.); 2 German Center for Infection Research (DZIF), Munich Site, 80336 Munich, Germany; 3 Institute of Virology, Campus Charite’ Mitte, Charite’-Universitätsmedizin Berlin, 10117 Berlin, Germany; anja.richter@charite.de (A.R.); christian.drosten@charite.de (C.D.); 4 Institute of Virology, School of Medicine, Technical University of Munich, 80333 Munich, Germany; andreas.pichlmair@tum.de; 5 Institute for Medical Microbiology, Virology and Hygiene, University Medical Center Hamburg-Eppendorf, 20246 Hamburg, Germany; s.pfefferle@uke.de; 6 Merck KGaA, 64293 Darmstadt, Germany; markus.b.klein@merckgroup.com (M.K.); ulrich.betz@merckgroup.com (U.A.K.B.); 7 Molecular Screening Shared Resource, California NanoSystems Institute (CNSI), The University of California, Los Angeles (UCLA), Los Angeles, CA 90095, USA; rdamoiseaux@mednet.ucla.edu

**Keywords:** LMP7, SARS-CoV-2, Nsp13, Nsp16, immunoproteasome, Ixazomib

## Abstract

The emergence of human coronaviruses has led to three epidemics or pandemics in the last two decades, collectively causing millions of deaths and thus highlighting a long-term need to identify new antiviral drug targets and develop antiviral therapeutics. In this study, a compound library was screened to uncover novel potential inhibitors of coronavirus replication. Three lead compounds, designated #16-14, #16-23, and #16-24, which were Ixazomib and its analogs, were identified based on their potent antiviral activity and minimal cytotoxicity. These compounds were found to inhibit the immunoproteasome subunit LMP7, a target whose subcellular localization and expression are altered in Severe Acute Respiratory Syndrome Coronavirus 2 (SARS-CoV-2)-infected Huh7 cells. Yeast two-hybrid assays and co-immunoprecipitation further revealed that LMP7 interacts with the viral proteins Nsp13 and Nsp16. In addition, Nsp13 and Nsp16 disrupted the expression of LMP7 in response to pathogen attacks. Functional studies showed that LMP7 knockout in BEAS-2B-ACE2 cells resulted in enhanced replication of attenuated SARS-CoV-2, highlighting the role of this subunit in restricting viral replication. Taken together, these findings position LMP7 as a novel therapeutic target and highlight Ixazomib and its analogs as potential antiviral agents against current and future coronavirus threats.

## 1. Introduction

The COVID-19 pandemic has lasted five years and resulted in more than seven million deaths worldwide [1]. Prior to the SARS-CoV outbreak in China in 2003, human coronaviruses were always considered to be mild viruses that cause cold-like symptoms. Over the past two decades, highly virulent coronaviruses have emerged every few years. Following the SARS-CoV outbreak, MERS-CoV was reported in 2012 and has so far caused 935 deaths, with a crude case fatality rate of 36% [2]. Just seven years later, SARS-CoV-2 was transmitted between humans and quickly became a pandemic. Therefore, the future emergence of novel human coronaviruses causing disease is a reasonable speculation, especially given the fact that several SARS-CoV- and SARS-CoV-2-related viruses are already known to exist in bats [3,4]. Although COVID-19 has moved from a pandemic emergency to an endemic state, the threat to public health from future coronaviruses remains. Further efforts are needed to discover new drug targets and develop new antiviral drugs.

Low-molecular-mass protein-7 (LMP7) is a catalytic subunit specifically present in the immunoproteasome induced under inflammatory conditions. The immunoproteasome is a special type of proteasome with a cylindrical structure composed of a 19S regulatory unit and a 20S proteolytic core complex [5,6]. The proteolytic core complex consists of two α outer rings and two β inner rings with seven subunits per ring. The two β inner rings contain three catalytically active subunits, PSMB6 (β1c), PSMB7 (β2c), and PSMB5 (β5c), in the classical constitutive proteasome, and these subunits are replaced by LMP2 (β1i), LMP10 (β2i), and LMP7 (β5i) in the immunoproteasome induced by proinflammatory cytokines.

The main function of the immunoproteasome is to process major histocompatibility complex (MHC) class I peptides for antigen presentation [7]. Previous research has demonstrated the anti-cancer role of immunoproteasome inhibitors. For example, the immunoproteasome-targeting compound PR-924 increases apoptosis in human hematological malignant cells and exhibits significant anti-leukemic properties [8]. Another inhibitor of the immunoproteasome that targets subunit LMP2, UK-101, was observed to induce apoptosis in PC-3 prostate cancer cells and inhibit tumor growth in vivo [9]. The antiviral activity of immunoproteasome inhibitors, including those that act against coronaviruses, is not yet understood. Ixazomib was the first oral proteasome inhibitor to be approved by the Food and Drug Administration (FDA) [10]. It has been shown to prolong progression-free survival in patients with multiple myeloma [11], but little is known about its potential role in antiviral therapy.

In this study, we identify Ixazomib and its analogs as novel LMP7 inhibitors that can significantly suppress coronavirus replication. LMP7 is targeted by SARS-CoV-2 viral proteins, and SARS-CoV-2 infection alters the subcellular distribution and protein levels of LMP7. Knockout of LMP7 significantly enhances the replication of the attenuated SARS-CoV-2 virus sCPD9. Our work suggests LMP7 as a potential drug target for treatments preventing human coronavirus replication.

## 2. Materials and Methods

### 2.1. Cell Culture and Transfection

HEK293, Huh7, and Human Angiotensin-converting enzyme 2 (ACE2)-RFP-transduced A549 cells (A549_ACE2_RFP) [12] were maintained in Dulbecco’s modified Eagle’s medium (DMEM, Gibco, ThermoFisher, Item Number: 11965092, Location: Waltham, MA, USA) supplemented with 10% fetal bovine serum (FBS) and 1% penicillin–streptomycin. BEAS-2B-ACE2 [13] cells were maintained in DMEM/F-12 (Gibco, ThermoFisher, Item Number: 11320033, Location: Waltham, MA, USA) containing 10% FBS, 1% penicillin–streptomycin, and 1% N-2-Hydroxyethylpiperazine-N-2-Ethane Sulfonic Acid (HEPES). Transfection of HEK293 cells was performed using Lipofectamine3000 (ThermoFisher, Item Number: L3000015, Location: Waltham, MA, USA) or 25 KD polyethyleneimine (PEI) according to the manufacturer’s protocols.

### 2.2. Plasmid and Constructions

GFP- and HA-fused LMP7 and RFP-fused HLA-A were generated by gateway cloning as described previously [14]. SARS-CoV-2 Nsp13 and Nsp16 were subsequently cloned into expression plasmids, following the method described recently in the literature [14].

For LMP7-knockout constructs, LMP7 sgRNAs were designed using the Synthego design tool: https://design.synthego.com/#/ (accessed on 2 October 2023)with the enzyme restriction sites of BsmbI. The oligo-dimers of LMP7 sgRNAs were cloned into a lentiCRISPR v2 vector, which was a gift from Feng Zhang (Addgene plasmid # 52961; http://n2t.net/addgene:52961, accessed on 22 July 2019; RRID: Addgene_52961) [15]. The packaging vector, psPAX2, was a gift from Didier Trono (Addgene plasmid # 12260; http://n2t.net/addgene:12260, accessed on 22 July 2019; RRID: Addgene_12260). pCMV-VSV-G was a gift from Bob Weinberg (Addgene plasmid # 8454; http://n2t.net/addgene:8454, accessed on 22 July 2019; RRID: Addgene_8454) [16].

### 2.3. Generation of LMP7-Knockout Cells

The successfully cloned pLentiCrispr v2 LMP7 was co-transfected with psPAX2 and pCMV-VSV-G plasmids into HEK293T cells. Three days after transfection, the lentivirus supernatant was harvested and BEAS-2B-ACE2 cells cultured in a 12-well plate were infected with this supernatant. Three days after infection, cells were selected using a growth medium containing 2 µg/mL puromycin for a period of between five and seven days. Surviving cells were harvested for Western blot analysis to determine the expression levels of LMP7.

### 2.4. Virus Infection

For a recombinant HCoV-229E virus expressing Renilla luciferase (HCoV-229E RLuc) infection, Huh7 or HEK293 cells transfected with aminopeptidase N (APN) receptors were infected with HCoV-229E RLuc (MOI = 1) [17]. At 24 h post-infection (24 h.p.i.), cells were harvested and replication of HCoV-229E RLuc was quantified using a Promega Renilla luciferase assay kit.

SARS-CoV-2 virus inhibition assays were performed using the IncuCyte S3 Live-Cell Analysis System (Essen Bioscience), as described in detail in [18]. A549_ACE2_RFP cells were infected with SARS-CoV-2-GFP at MOI = 1. The GFP levels were quantified every 4 h until 72 h.p.i. Cell confluency was measured in parallel as a measure of cell viability.

For infection with the attenuated SARS-CoV-2 virus sCPD9 [19,20], BEAS-2B-ACE2 cells were challenged with sCPD9 (MOI = 0.0001). Two hours after infection, the cells were washed with DPBS and supplied with fresh growth medium. Five days after infection, the cells were harvested for RNA isolation. Viral RNAs were quantified by qPCR using primers and probes specific to the SARS-CoV-2 nucleocapsid protein.

For vaccinia virus vTF-7 infection, HEK293 cells were infected with a recombinant vaccinia virus, vTF-7, expressing the T7 RNA polymerase as described previously [21]. At 24 h.p.i., cells were harvested for immunofluorescence staining or Western blot analysis.

### 2.5. Cell Viability Assay

The viability of the cells was determined using a CellTiter-Glo^®^ 2.0 Cell Viability Assay (Promega, Item Number: #G9242, Location: Madison, WI, USA). The cell lines were plated in 96-well plates and incubated with inhibitor concentrations corresponding to each inhibition experiment.

### 2.6. Immunofluorescence Staining and Fluorescence Microscopy

Immunofluorescence staining and fluorescence microscopy were performed as previously described [14]. Briefly, mock and infected cells were seeded on coverslips in a 24-well plate, fixed with 4% paraformaldehyde (PFA) in PBS for 15 min at room temperature, and washed with PBS. Cell membranes were permeabilized by 0.1% Triton X-100 in PBS for 15 min. The cells were washed again with PBS and blocked in PBS containing 5% bovine serum albumin (BSA) and 0.2% Tween-20 for one hour at room temperature. The cells were then incubated with primary antibodies diluted in PBS containing 5% BSA and 0.2% Tween-20 at 4 °C overnight. After washing with PBS and incubation with secondary antibodies for one hour in the dark, the cells were washed and stained with DAPI diluted to 1:1000 in PBS for 10 min in the dark and mounted on glass slides. Images were captured using a Leica DM4000 B fluorescence microscope with a 40× objective.

### 2.7. Co-Immunoprecipitation and Western Blot Analysis

HEK293 cells were transfected with plasmids in a 6-well plate using Lipofectamine 3000 (Thermofisher). Twenty-four or forty-eight hours after transfection, cells were lysed and their protein was purified with GFP-Trap_A bead-based co-immunoprecipitation (Co-IP) (ChromoTek, Planegg-Martinsried, Germany) according to the manufacturer’s protocol. The Western blot protocol has been described elsewhere [22]. The details of antibodies used in Western blot are described in Table S2.

### 2.8. Statistics

Statistical analysis was performed using one-way analysis of variance (one-way ANOVA) with Dunnett’s test for multiple comparisons or Student’s *t*-test for two-group analysis using GraphPad Prism 10.5.0 software with the significance level set at α = 0.05. Symbols in the figures represent *p*-values: ns indicates not significant and *p* ≥ 0.05, * indicates 0.01 < *p* < 0.05, ** indicates 0.001 < *p* < 0.01, and *** indicates *p* < 0.001.

## 3. Results

### 3.1. Coronavirus Replication Inhibitors Selected from Screening Are LMP7 Inhibitors

**Figure 1:**
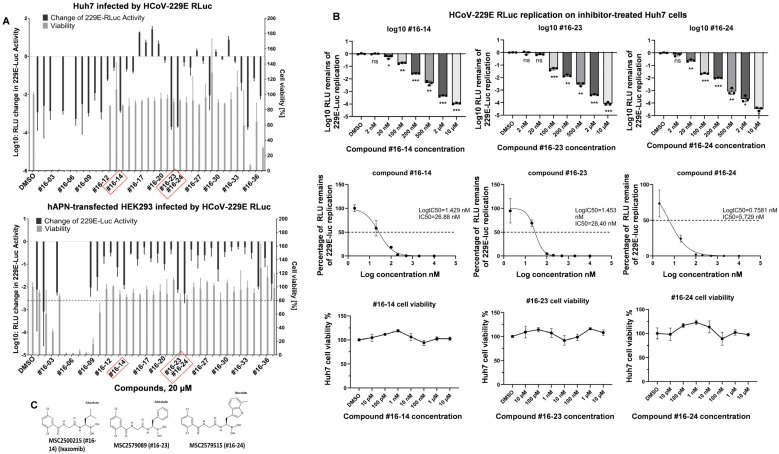
The drugs selected from the screening to inhibit coronavirus replication are LMP7 inhibitors. (**A**) For the inhibition screen, Huh7 cells and HEK293 cells transiently expressing hAPN in 96-well plates were inoculated with HCoV-229E RLuc (MOI = 1) at 33 °C for 1 h. After inoculation, the cells were washed with PBS and cultured in a growth medium containing each compound for 24 h before harvesting for luciferase activity measurements. For the cell viability assays, Huh7 and HEK293 cells in 96-well plates were treated with the indicated individual compound for 24 h before cell viability was measured using a CellTiter-Glo^®^ 2.0 Cell Viability Assay Kit (Promega). Means and standard deviations were calculated based on three biological replicates (*n* = 3) for both the inhibition screening and cell viability assays. Candidate compounds were selected according to their cell viability (more than 80%) and inhibition rates (below 0.1% for Huh7 cells and below 1% for HEK293 cells). (**B**) For the inhibition assays, Huh7 cells in 96-well plates were infected with HCoV-229E RLuc (MOI = 1) and treated with the indicated inhibitors for luciferase activity measurement as described above. Three biological replicates were performed for each compound concentration to calculate the means and standard deviations for the graphs (*n* = 3). Inhibition curves and corresponding logIC50 and IC50 values were generated using GraphPad Prism. Cell viability assays were performed as described above with three biological replicates (*n* = 3). * indicates *p* < 0.05, ** indicates *p* < 0.01, *** indicates *p* < 0.001 and ns indicates not significant. (**C**) The structural formulas of the inhibitors (#16-14 (Ixazomib), #16-23, and #16-24).

To identify new drug candidates that suppress coronavirus replication, the recombinant human coronavirus HCoV-229E RLuc, carrying the Renilla luciferase reporter [23], was used to rapidly screen a compound library. As a result, three compounds (#16-14, #16-23, and #16-24) were selected due to their potent antiviral activity and low cytotoxicity in both Huh7 cells and HCoV-229E receptor APN-transfected HEK293 cells (Figure 1A). Detailed HCoV-229E RLuc inhibition assays in Huh7 cells showed that the IC50 values of compounds #16-14, #16-23, and #16-24 are 26.88 nM, 28.40 nM, and 5.729 nM, respectively (Figure 1B). Interestingly, all three selected compounds are LMP7 inhibitors with remarkable inhibition efficiencies for LMP7 in cell-free biochemical assays [24] (Table 1). Compound #16-14 is the well-known drug Ixazomib. Compounds #16-23 and #16-24 are analogs of Ixazomib. Their structural formulas are shown in Figure 1C.

### 3.2. Candidate Compounds Inhibit Replication of SARS-CoV and SARS-CoV-2

**Figure 2:**
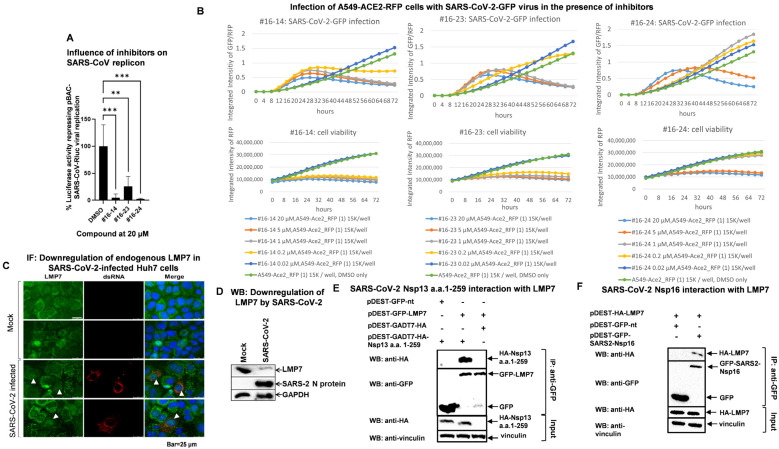
The effects of the candidate compounds on coronavirus replication and LMP7 expression during SARS-CoV-2 infection. (**A**) HEK293 cells seeded in a 96-well plate were transfected with a pBAC-SARS-CoV replicon carrying a Renilla luciferase reporter (pBAC-SARS-CoV-Rluc). Four hours after transfection, either DMSO (control) or the individual compound was added to the growth medium, and the cells were cultured for 24 h before luciferase activity was measured. Means and standard deviations were calculated based on four biological replicates (*n* = 4). Statistical analysis was performed using one-way ANOVA. ** indicates *p* < 0.01, and *** indicates *p* < 0.001. (**B**) A549-ACE2-RFP cells were infected with the SARS-CoV-2-GFP virus (MOI = 1), and the intensity of the GFP signals was measured every 4 h until 72 h after infection. Cell confluences were assessed simultaneously as a measure of cell viability. SARS-CoV-2 virus inhibition assays were performed using an IncuCyte S3 Live-Cell Analysis System (Essen Bioscience), as described in detail [18]. (**C**) SARS-CoV-2 infection downregulates endogenous LMP7 in immunofluorescence (IF) staining. Huh7 cells seeded on coverslips in a 24-well plate were either mock-treated or infected with SARS-CoV-2 (MOI = 0.1; B.1 strain EPI_ISL_406862) for 24 h before fixation. Anti-LMP7 and anti-dsRNA antibodies were used for IF staining. Infection and staining were performed in duplicate. White arrows: endogenous LMP7. (**D**) SARS-CoV-2 infection reduces endogenous LMP7 levels in Huh7.5 cells. Cells seeded in a 6-well plate were either mock-treated or infected with SARS-CoV-2 (MOI = 1, 48 h) before harvesting for Western blot analysis. N: nucleocapsid. (**E**) LMP7 interacts with SARS-CoV-2 Nsp13 a.a.1-259 in HEK293 cells. Cells in a 6-well plate were transfected with the indicated plasmids. Then, 2.5 h after transfection, the cells were infected with the vaccinia vTF-7 virus to induce expression of the T7 promotor-driven pDEST-GADT7-HA-Nsp13 a.a. 1-259. After a further 48 h, the cells were harvested for GFP-trap-based CoIP and analyzed by Western blot with the indicated antibodies. (**F**) LMP7 interacts with SARS-CoV-2 Nsp16. HEK293 cells in a 6-well plate were transfected with the indicated plasmids. Then, 48 h after transfection, the cells were harvested for GFP-trap-based CoIP and analyzed by Western blot with the indicated antibodies.

The selected compounds were then tested for their ability to inhibit the replication of highly virulent human coronaviruses. A SARS-CoV replicon carrying the Renilla luciferase reporter pBAC-REP-RLuc [25,26], which mimics SARS-CoV replication, was transfected into HEK293 cells with or without post-transfection drug treatment. As shown in Figure 2A, all selected compounds significantly inhibited the replication of this SARS-CoV replicon.

The compounds were also tested for their ability to suppress SARS-CoV-2 replication. A549_ACE2_RFP cells were challenged with a recombinant SARS-CoV-2 virus carrying a GFP reporter (SARS-CoV-2-GFP) [18] in the presence of the compounds. The ratio of GFP and RFP intensity, representing viral spread and replication, was measured every 4 h for 72 h. The RFP intensity of uninfected cells treated with the drugs was measured to assess the cytotoxicity of the drugs. As shown in the lower graphs in Figure 2B, the RFP intensity remained constant with a high-concentration drug treatment and even increased with a low-concentration drug treatment. This indicates the drugs have low cytotoxicity in A549_ACE2_RFP cells such that no cells died after 72 h of drug treatment. The upper graphs in Figure 2B reveal that all compounds inhibited viral growth of SARS-CoV-2-GFP, although less efficiently than HCoV-229E-RLuc in Huh7 cells when compared to Figure 1B.

### 3.3. SARS-CoV-2 Infection Reduces LMP7 Protein Levels, and SARS-CoV-2 Viral Proteins Target LMP7

We then investigated whether SARS-CoV-2 infection alters the subcellular localization or protein level of LMP7. Huh7 cells infected with SARS-CoV-2 at an MOI of 0.1 for 24 h were fixed and examined by immunofluorescence microscopy. As shown in Figure 2C and Figure S1, in uninfected Huh7 cells, LMP7 was mainly located in the cytosol, although it was occasionally found in the nucleus. However, in SARS-CoV-2-infected Huh7 cells, as indicated by dsRNA staining, LMP7 was mainly located in the nucleus and normally showed low expression. To confirm that SARS-CoV-2 infection reduced LMP7 expression, Western blot analysis was performed on Huh7.5 cells. Huh7.5 is more susceptible to SARS-CoV-2 than Huh7 and therefore always displays a stronger and more convincing phenotype upon infection. As shown in Figure 2D, SARS-CoV-2 infection caused a significant reduction in the expression of LMP7.

To further investigate how LMP7 is regulated by SARS-CoV-2, we used LMP7 as a bait to screen SARS-CoV-2 viral proteins in yeast two-hybrid (Y2H) assays [14]. As a result, LMP7 was found to interact with SARS-CoV-2 Nsp13 a.a.1-259 and Nsp16 (Figure S2). The interactions were then verified in mammalian cells by Co-IP. As Nsp13 a.a.1-259 showed relatively weak expression under the control of the CMV promoter, pDEST-GADT7 Nsp13 a.a.1-259, in which Nsp13 a.a.1-259 is under the control of the T7 promoter, was used. After induction with the vaccinia vTF-7 virus, optimal expression of Nsp13 a.a.1-259 was achieved. As shown in Figure 2E, SARS-CoV-2 Nsp13 a.a.1-259 clearly interacted with LMP7 upon vaccinia vTF-7 stimulation. In addition, the interaction between SARS-CoV-2 Nsp16 and LMP7 was also confirmed in HEK293 cells by Co-IP (Figure 2F).

### 3.4. SARS-CoV-2 Nsp13 and Nsp16 Inhibit LMP7 Expression, and Nsp16 Disrupts LMP7-Mediated Antigen Presentation

**Figure 3:**
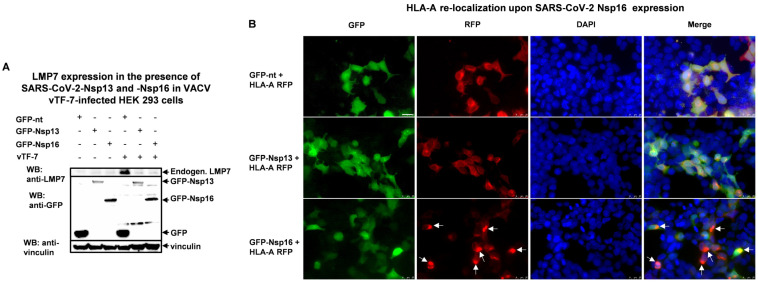
The influence of SARS-CoV-2-Nsp13 and -Nsp16 on LMP7 expression and perturbation of HLA-A expression by Nsp16. (**A**) SARS-CoV-2 Nsp13 and Nsp16 suppress the expression of endogenous LMP7 in response to infection with the vaccinia virus vTF-7 (expressing T7 polymerase). HEK293 cells seeded in a 12-well plate were transfected with plasmids expressing a pDEST-GFP control (GFP-nt), pDEST-GFP-SARS-CoV-2-Nsp13, or pDEST-GFP-SARS-CoV-2-Nsp16. After 3.5 h, the cells were infected with vTF-7 to induce a host antiviral response and incubated for 24 h before harvesting for WB analysis. (**B**) SARS-CoV-2 Nsp16 interferes with HLA-A antigen presentation (HEK293, vTF-7, 24 h). HEK293 cells seeded in a 24-well plate with coverslips were transfected with plasmids expressing HLA-A-RFP and -GFP controls, GFP-SARS-CoV-2 Nsp13, or GFP-SARS-CoV-2 Nsp16. Then, 3.5 h post-transfection, the cells were challenged with VACV and incubated for 24 h before fixation and subsequent DAPI staining. The white arrows indicate the cells with altered HLA-A-RFP subcellular localization after co-transfection with GFP-Nsp16. The scale bar represents 25 µm.

The vaccinia virus has been shown to infect HEK293 cells and induce an antiviral host response [27]. Therefore, we infected HEK293 cells with the vaccinia virus vTF-7 (VACV) and observed an increase in endogenous LMP7 expression compared to the uninfected control group (Figure 3A, compare lane 1 and lane 4). This stimulation was inhibited when Nsp13 a.a.1-259 or Nsp16 was transfected.

LMP7 is a catalytic subunit of the immunoproteasome, whose primary function is to process peptides for antigen presentation via MHC class I. During the process of antigen presentation, MHC class I migrates from the endoplasmic reticulum (ER) to the cell membrane to expose the bound peptide to T-cell receptors [28]. To further investigate the effect of these two SARS-CoV-2 proteins on LMP7 function in relation to antigen presentation, we co-transfected HEK293 cells with GFP fused to Nsp13 or Nsp16 and RFP fused to HLA-A, HLA-B, and HLA-C, which are major members of the MHC class I heavy chain paralogs, and then infected the cells with the vaccinia virus vTF-7 for 24 h. Figure 3B and Figure S3 demonstrate that GFP-Nsp16 altered the subcellular localization of HLA-A, HLA-B, and HLA-C compared to an empty-vector group. We speculate that Nsp16 interferes with the process of antigen presentation mediated by LMP7.

### 3.5. LMP7 Is an Antiviral Factor That Acts Against SARS-CoV-2

**Figure 4:**
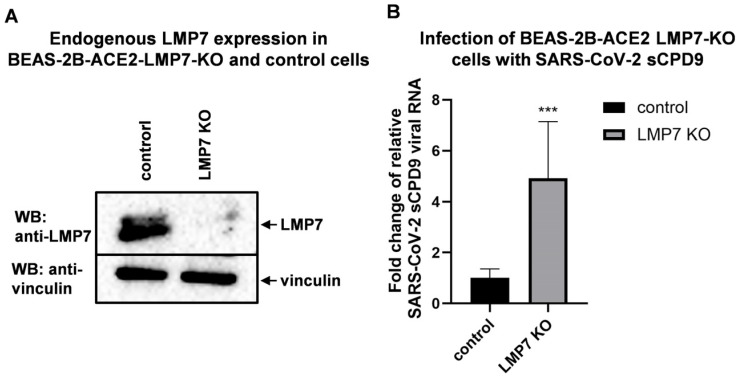
LMP7 is an antiviral factor that acts against SARS-CoV-2. (**A**) Western blot analysis of the endogenous LMP7 levels in BEAS-2B-ACE2-LMP7-knockout (LMP7-KO) and control cells. (**B**) BEAS-2B-ACE2-LMP7-KO and control cell lines were infected with SARS-CoV-2 SCPD9 (MOI = 0.0001) in a 24-well plate. At 2 h post-infection, the cells were washed twice with DPBS and replenished with fresh growth medium. Then, 5 days post-infection, the cells were harvested for RNA isolation, followed by qPCR using virus-specific primers and probes. The mRNA levels were normalized using β-actin. Means and standard deviations were calculated based on three biological replicates (*n* = 3). Statistical analysis was performed using Student’s *t*-test. *** indicates *p* < 0.001.

To further understand the role of LMP7 in human coronavirus replication, LMP7 was knocked out in human ACE2-transgenic bronchial epithelial cells (BEAS-2B-ACE2), as this cell line shows abundant endogenous LMP7 expression. In addition, this cell line is susceptible to infection with SARS-CoV-2 and mild coronaviruses. The endogenous protein level of LMP7 was greatly reduced in the LMP7-knockout (BEAS-2B-ACE2 LMP7-KO) cells (Figure 4A) compared to the empty-vector control (BEAS-2B-ACE2 control). After infection with the attenuated SARS-CoV-2 virus sCPD9, cells that were deficient in LMP7 produced more virus (Figure 4B).

## 4. Discussion

LMP7 is a catalytic subunit of an immunoproteasome that degrades viral protein into peptides during infection for downstream antigen presentation at the cell surface. In this way, infected cells can be recognized by CD8+ T cells. In our study, we demonstrated that LMP7 negatively regulates SARS-CoV-2 replication, as LMP7 knockout promoted viral growth (Figure 4B). This indicates that LMP7 is vital for proteasome activity and subsequent antigen presentation. To antagonize LMP7 or degradation by immunoproteasomes, SARS-CoV-2 Nsp13 and Nsp16 bind to (Figure 2E,F) and decrease the expression of (Figure 3A) LMP7. As a consequence, expression of Nsp16 disturbs antigen presentation by altering the subcellular localization of MHC class I molecules (Figure 3B and Figure S3A,B). But it is not clear why expression of Nsp13 does not lead to an altered subcellular distribution upon viral infection.

LMP7 has been shown to be an antiviral factor in several research studies. During rhinovirus infection, airway epithelial immunoproteasome subunit LMP7 was observed to exert anti-inflammatory and antiviral effects, thereby facilitating the resolution of inflammation and a reduction in the viral load [29]. In addition, LMP7 can interact with and positively regulate the expression of A20, which can fight respiratory viral infections such as the influenza A virus [30]. These findings also provide potential explanations for why LMP7 negatively regulates coronavirus replication.

Our study demonstrated that LMP7 deficiency promotes SARS-CoV-2 replication (Figure 4B). However, the compound Ixazomib and its analogs act as LMP7 inhibitors (Table 1) and clearly suppress coronavirus replication (Figure 1B and Figure 2A,B). The reason for this pseudo-contradiction is that the pharmaceutical mechanism of these compounds is very likely independent from LMP7, although they can also inhibit LMP7 activity. It has been shown that Ixazomib is not only an LMP7-specific inhibitor; it can also inhibit other subunits of the immunoproteasome such as LMP2 and some subunits of the constitutive classic proteasome [24]. The proteasome inhibitors MG132, epoxomicin, and Velcade (bortezomib) have shown clear coronavirus-inhibitory activity in cell culture, mainly by affecting the early steps of coronavirus infection [31]. It has been demonstrated that functional proteasome–ubiquitin systems facilitate the release of coronavirus from endosomes into the cytosol after viral entry, as pretreatment with MG132 can retain murine coronavirus (murine hepatitis virus, MHV) in endosomes after virus internalization into cells [32,33]. Besides immunoproteasomes, Ixazomib also exhibits inhibitory activity against constitutive classic proteasomes [24], similar to MG132. It can be speculated that Ixazomib and its analogs act similarly to MG132 to effectively restrict coronavirus release from endosomes into the cytosol after viral entry through endocytosis. This mechanism is independent of LMP7 and immunoproteasome inhibition and should be the main pharmaceutical mechanism for Ixazomib and its analogs to inhibit coronavirus replication, as this is a very early step after viral entry, before the virus unpacks to release structural proteins and starts to express nonstructural proteins in the cytosol for degradation by immunoproteasomes. In addition to endocytosis, the coronavirus can also enter host cells via direct membrane fusion [34], preventing it from being retained in endosomes by proteasome inhibitors. But if this is the major case, inhibition of LMP7 by our compounds should not lead to a reduction in viral replication.

Antigen presentation mediated by MHC class I molecules is downstream of immunoproteasomes and is a key process in the immune response to SARS-CoV-2 infection, influencing both viral replication and disease progression. The SARS-CoV-2 virus shows a high capacity to suppress the induction of MHC-I in infected cells in vivo [35]. The SARS-CoV-2 accessory protein ORF7a has been observed to interact specifically with the MHC-I heavy chain, thereby competing with the binding of the small protein β2-microglobulin. This interaction has been shown to reduce antigen presentation by the human MHC-I allele HLA-A*02:01 [36]. It is not surprising that Nsp16, which decreases LMP7, also disturbed the distribution of HLA proteins upon viral infection in our study. But whether Nsp16 affects the subsequent process, such as activation of antigen-specific CD8+ T cells, requires further investigation. Furthermore, we observed that Nsp13 did not alter the subcellular localization of HLA-A, HLA-B, and HLA-C. Nevertheless, we cannot rule out the possibility that Nsp13 affects other stages of the MHC-I antigen presentation pathway, such as peptide loading or transport to the cell surface [37]. This needs further exploration.

### Limitations of This Study

One of the limitations of this study is the unresolved paradox between the antiviral effects of pharmacological inhibition of LMP7 and the proviral phenotype observed upon LMP7 knockout. Our data suggest that Ixazomib and its analogs likely inhibit coronavirus replication through a mechanism independent of LMP7. However, direct experimental evidence supporting this proposed off-target, immunoproteasome-independent mechanism is currently limited. Future studies are needed to precisely define the molecular basis of this early-stage antiviral activity and to further distinguish between LMP7-specific and LMP7-independent pathways. Additionally, while we used multiple human cell lines to evaluate the consistency of the observed phenotypes, we acknowledge that the use of different cell types with varying levels of susceptibility introduces some degree of variability. Future studies using more physiologically relevant models, such as primary cells or organoids, will be valuable to further validate these findings. Another limitation of this study is the lack of in vivo validation of the antiviral efficacy of Ixazomib and its analogs. While our in vitro results demonstrate potent inhibition of multiple human coronaviruses, including SARS-CoV-2, further studies in appropriate animal models, such as SARS-CoV-2-infected hamsters or transgenic mice, are necessary to confirm the therapeutic potential and safety profile of these compounds in vivo.

## 5. Conclusions

In summary, our study uncovered the role of LMP7 during SARS-CoV-2 infection. LMP7 deficiency promotes viral replication, indicating that LMP7 is a restriction factor for SARS-CoV-2. To counteract this restriction factor, SARS-CoV-2 Nsp13 and Nsp16 interact with LMP7, and expression of Nsp13 or Nsp16 reduces LMP7 at the protein level upon infection. Additionally, Nsp16 alters the subcellular localization of MHC class I molecules responsible for antigen presentation during infection. In parallel, three compounds, #16-14 (Ixazomib), #16-23, and #16-24, are LMP7 inhibitors and can effectively suppress coronavirus replication. But their pharmaceutical mechanism should be LMP7-independent.

## Figures and Tables

**Table 1:** IC50 values of immunoproteasome subunit LMP7 inhibitors #16-14 (Ixazomib), #16-23, and #16-24 in a cell-free biochemical assay [24] and in HCoV-229E-infected Huh7 cells.

Inhibitor ID	IC50 (nM) of LMP7 in Cell-Free Biochemical Assay	IC50 (nM) of 229E-Rluc Inhibition in Huh7 Cells
#16-14	2.88	26.88
#16-23	1.1	28.40
#16-24	0.3	5.73

## Data Availability

The data is contained within this article or the Supplementary Materials.

## Supplementary Materials

The following supporting information can be downloaded at https://www.mdpi.com/article/10.3390/pathogens14090871/s1, Figure S1: SARS-CoV-2 infection downregulates endogenous LMP7 in immunofluorescence (IF) staining, Figure S2: The interaction between LMP7 and SARS-CoV-2 proteins in Y2H assays, Figure S3: HLA-B and HLA-C re-localization upon SARS-CoV-2 Nsp16 expression; Table S1: Primer list, Table S2: Antibody list.
