# Supplementary material for: LMP7 as a Target for Coronavirus Therapy: Inhibition by Ixazomib and Interaction with SARS-CoV-2 Proteins Nsp13 and Nsp16
Source: Pathogens. 2025 Sep 2;14(9):871. doi: 10.3390/pathogens14090871 (PMC12472737; doi:10.3390/pathogens14090871)
Supplement: Supplementary file 1 [file pathogens-14-00871-s001.zip › supplementary table 2 antibody list.pdf]

Table S2 Antibody list

target	Strain, company	dilution	source
anti-vincullin	V9264, Sigma	1:1000	mouse
anti-GFP	A6455, invitrogen	1:1000-1500	rabbit
anti-HA	Clone 3F10, Roche	1:1000	rat
anti-SARS-CoV / SARS-CoV-2-N	GTX632269(6H3), GeneTeX	1:1000	mouse
anti-GAPDH	Sc-365062, Santa cruz Biotechnology	1:2000	mouse
anti-LMP7	14859-1-AP, Proteintech	1:1000	rabbit
anti-dsRNA	J2, SCICONS	1:1000	mouse
anti-rabbit	P0217, DAKO	1:1000	
anti-mouse	A9917, sigma	1:10 000	
anti-rat	A9037, sigma	1:5000	
