# Supplementary material for: LMP7 as a Target for Coronavirus Therapy: Inhibition by Ixazomib and Interaction with SARS-CoV-2 Proteins Nsp13 and Nsp16
Source: Pathogens. 2025 Sep 2;14(9):871. doi: 10.3390/pathogens14090871 (PMC12472737; doi:10.3390/pathogens14090871)
Supplement: Supplementary file 1 [file pathogens-14-00871-s001.zip › supplementary table 1 primer list.pdf]

Table S1 Primer list

Primer name	Sequence (5′–3′)
β-actin qPCR F	TCCTGAGCGCAAGTACTCCG
β-actin qPCR R	CTGATCCACATCTGCTGGAAGG
β-actin qPCR probe	6-FAM-ATCGGCGGCTCCATCCTG-BHQ1
SARS-CoV-2 qPCR F	GACCCCAAAATCAGCGAAAT
SARS-CoV-2 qPCR R	TCTGGTTACTGCCAGTTGAATCTG
SARS-CoV-2 qPCR probe	6-FAM-ACCCCGCATTACGTTTGGTGGACC-BHQ1
