# Supplementary material for: LMP7 as a Target for Coronavirus Therapy: Inhibition by Ixazomib and Interaction with SARS-CoV-2 Proteins Nsp13 and Nsp16
Source: Pathogens. 2025 Sep 2;14(9):871. doi: 10.3390/pathogens14090871 (PMC12472737; doi:10.3390/pathogens14090871)
Supplement: Supplementary file 1 [file pathogens-14-00871-s001.zip › supplementary figures.pdf]

Figure S1

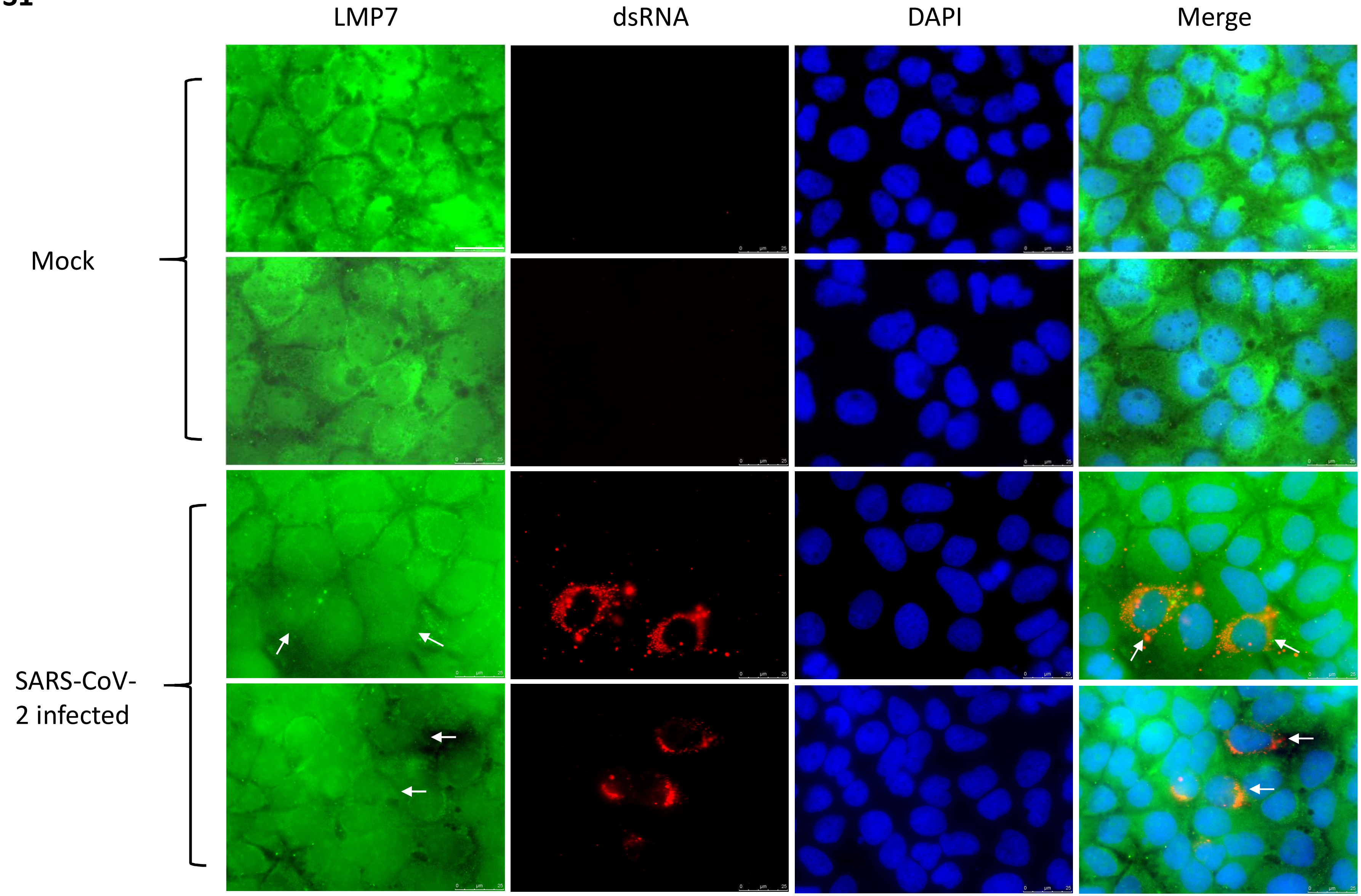

Legend to Figure S1

Figure S1. SARS-CoV-2 infection down-regulates endogenous LMP7 in immunofluorescence (IF) staining. Huh7 cells were cultured on coverslips placed in a 24-well plate and subjected to either mock treatment or infection with SARS-CoV-2 (MOI=0.1; B.1 strain EPI_ISL_406862) for 24 hours prior to fixation. Immunofluorescence staining was performed using anti-LMP7 and anti-dsRNA antibodies. Two more fields were chosen randomly for mock and infection groups. Scale bar: 25 µm.

Figure S2

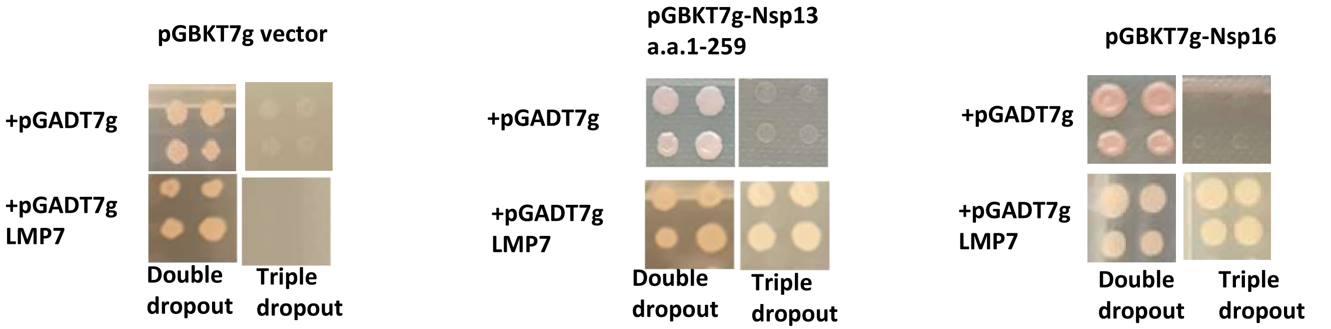

pGBKT7g vector
+pGADT7g
+pGADT7g
LMP7
Double
dropout
Triple
dropout
pGBKT7g-Nsp13
a.a.1-259
+pGADT7g
+pGADT7g
LMP7
Double
dropout
Triple
dropout
pGBKT7g-Nsp16
+pGADT7g
+pGADT7g
LMP7
Double
dropout
Triple
dropout

Legend to Figure S2

Figure S2. The pGADT7g vector encoding LMP7 or an empty control vector was co-transformed into competent yeast PJ69–7A cells along with pGBKT7g plasmids expressing individual SARS-CoV-2 genes. Successful co-transformation was verified by the growth of yeast colonies on double dropout agar plates. Interaction between LMP7 and viral proteins was indicated by colony growth on triple dropout agar plates, specifically demonstrating interactions between LMP7 and Nsp13 (amino acids 1–259; middle panel), as well as between LMP7 and Nsp16 (right panel). No non-specific interaction was detected between LMP7 and the empty pGBKT7g vector (left panel).

Figure S3A

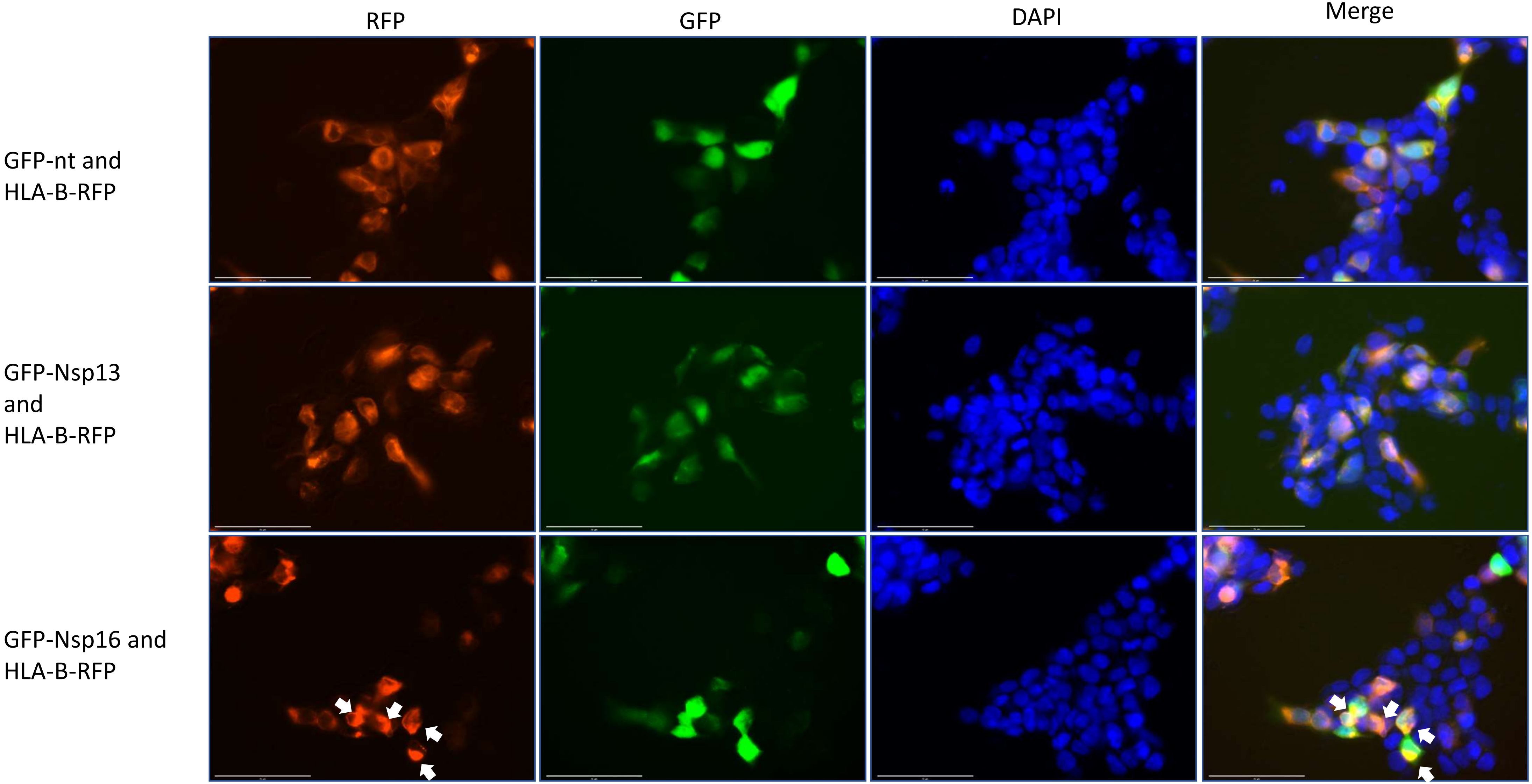
RFP
GFP
DAPI
Merge
GFP-nt and
HLA-B-RFP
GFP-Nsp13
and
HLA-B-RFP
GFP-Nsp16 and
HLA-B-RFP

Figure S3B

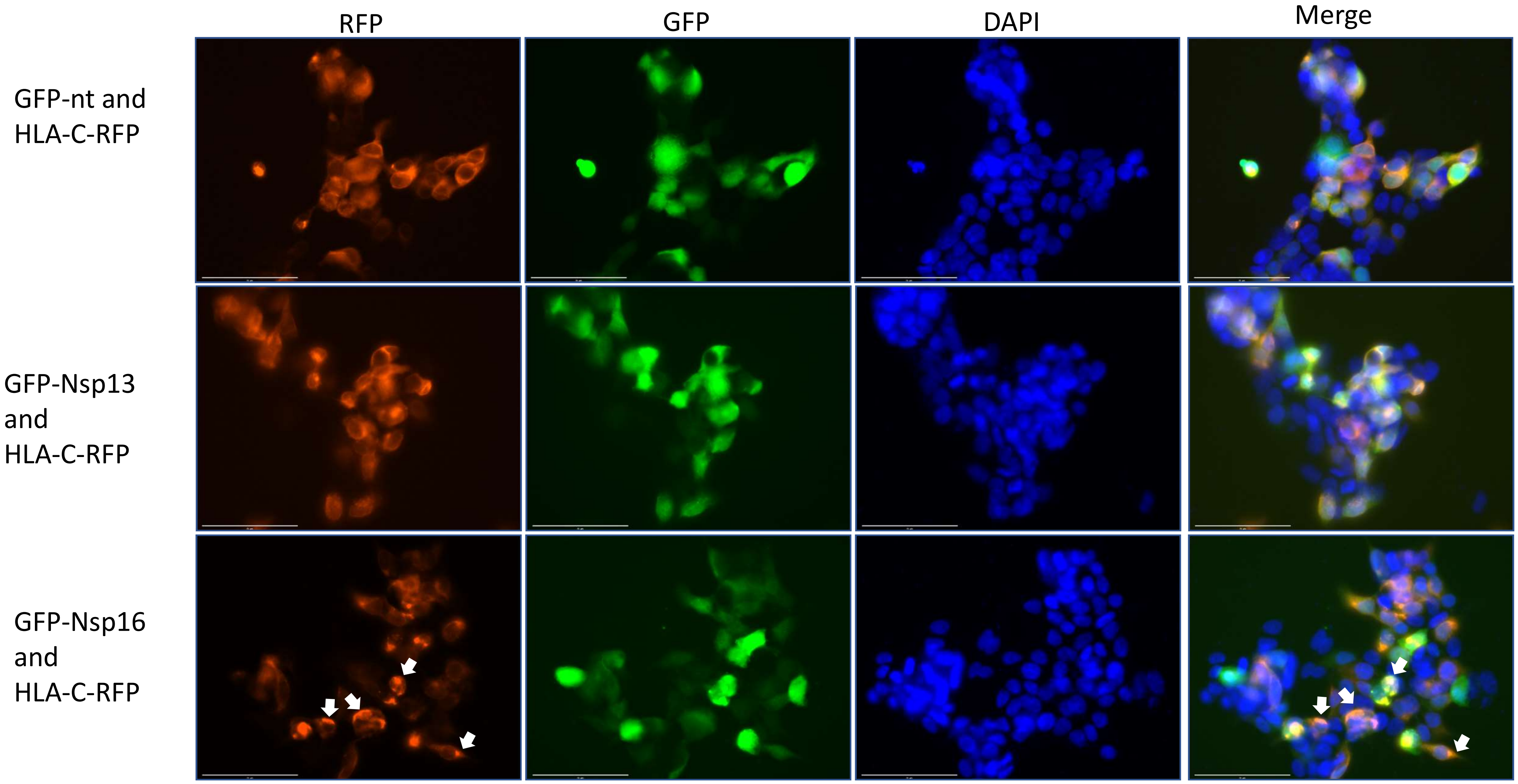
RFP
GFP
DAPI
Merge
GFP-nt and HLA-C-RFP
GFP-Nsp13 and HLA-C-RFP
GFP-Nsp16 and HLA-C-RFP

Legend to Figure S3

Figure S3. HEK293 cells were transfected with GFP-target proteins and HLA-B-RFP (A) or HLA-C-RFP (B) plasmids in 24 well plates. Cells were infected with vaccinia virus vTF-7. Forty-eight hours after transfection, cells were fixed with 4% PFA and stained with DAPI. Three fields were chosen randomly. Scale bar: 75 µm.
